# Tetra­aqua­bis­(2-methyl-1*H*-imidazole-κ*N* ^3^)cobalt(II) naphthalene-1,5-disulfonate

**DOI:** 10.1107/S160053681104548X

**Published:** 2011-11-05

**Authors:** Yu Jin

**Affiliations:** a Ordered Matter Science Research Center, Southeast University, Nanjing 211189, People’s Republic of China

## Abstract

In the title complex, [Co(C_4_H_6_N_2_)_2_(H_2_O)_4_](C_10_H_6_O_6_S_2_), the cation and anion both reside on crystallographic inversion centers, such that the asymmetric unit comprises one half cation and one half anion. The central Co^II^ ion is coordinated by four water mol­ecules and two 2-methyl­imidazole ligands, resulting in a *trans*-octa­hedral coordination geometry. The existence of strong N—H⋯O and O—H⋯O hydrogen-bonding inter­actions gives rise to a three-dimensional structure.

## Related literature

For general background to ferroelectric metal-organic frameworks, see: Wu *et al.* (2011 ▶); Ye *et al.* (2006 ▶); Zhang *et al.* (2008 ▶, 2010) ▶; Fu *et al.* (2009 ▶). 
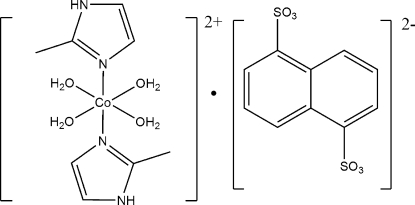


## Experimental

### 

#### Crystal data

[Co(C_4_H_6_N_2_)_2_(H_2_O)_4_](C_10_H_6_O_6_S_2_)
*M* *_r_* = 581.48
Monoclinic, 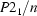

*a* = 8.0260 (16) Å
*b* = 12.923 (3) Å
*c* = 11.658 (2) Å
β = 99.27 (3)°
*V* = 1193.5 (4) Å^3^
*Z* = 2
Mo *K*α radiation
μ = 0.96 mm^−1^
*T* = 293 K
0.3 × 0.3 × 0.2 mm

#### Data collection

Rigaku Mercury CCD diffractometer
Absorption correction: multi-scan (*CrystalClear*; Rigaku, 2005 ▶) *T*_min_ = 0.489, *T*_max_ = 1.000
12041 measured reflections
2729 independent reflections
2558 reflections with *I* > 2σ(*I*)
*R*_int_ = 0.031

#### Refinement

*R*[*F* ^2^ > 2σ(*F* ^2^)] = 0.028
*wR*(*F* ^2^) = 0.073
*S* = 1.09
2729 reflections
177 parameters
4 restraints
H atoms treated by a mixture of independent and constrained refinement
Δρ_max_ = 0.30 e Å^−3^
Δρ_min_ = −0.27 e Å^−3^

### 

Data collection: *CrystalClear* (Rigaku, 2005 ▶); cell refinement: *CrystalClear*; data reduction: *CrystalClear*; program(s) used to solve structure: *SHELXS97* (Sheldrick, 2008 ▶); program(s) used to refine structure: *SHELXL97* (Sheldrick, 2008 ▶); molecular graphics: *SHELXTL* (Sheldrick, 2008 ▶); software used to prepare material for publication: *SHELXL97*.

## Supplementary Material

Crystal structure: contains datablock(s) I, global. DOI: 10.1107/S160053681104548X/fj2459sup1.cif

Structure factors: contains datablock(s) I. DOI: 10.1107/S160053681104548X/fj2459Isup2.hkl

Additional supplementary materials:  crystallographic information; 3D view; checkCIF report

## Figures and Tables

**Table 1:** Hydrogen-bond geometry (Å, °)

*D*—H⋯*A*	*D*—H	H⋯*A*	*D*⋯*A*	*D*—H⋯*A*
O2—H4⋯O5^i^	0.85 (1)	1.97 (1)	2.815 (2)	174 (3)
O2—H5⋯O4^ii^	0.84 (1)	1.88 (1)	2.7149 (19)	171 (3)
O1—H6⋯O5^ii^	0.84 (1)	2.21 (1)	3.026 (2)	167 (3)
O1—H7⋯O3^iii^	0.84 (1)	1.92 (1)	2.7661 (18)	179 (2)
N1—H1*B*⋯O4	0.86	2.06	2.906 (2)	170

Symmetry codes: (i) 
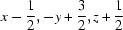
; (ii) 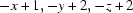; (iii) 
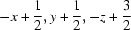
.

**Fig. 1.**
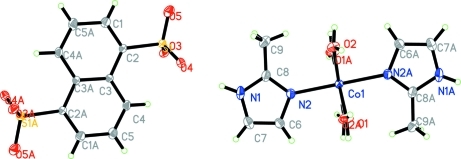
Crystal structure of the title compound with labelling and displacement ellipsoids drawn at the 30% probability level.

**Fig. 2.**
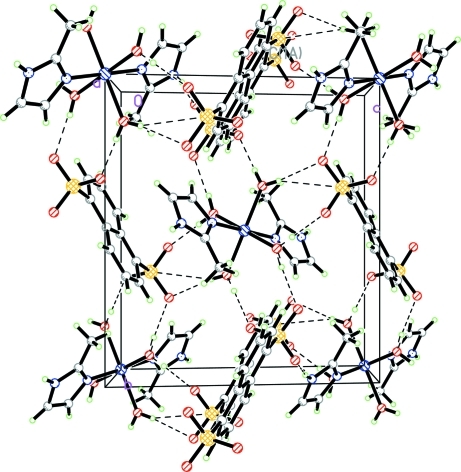
Crystal structure of the title compound with view along the a axis. Intermolecular interactions are shown as dashed lines.

## supplementary crystallographic information

### Comment

In recent years, simple molecular-ionic compounds containing inorganic cations and organic anions have attracted great interest owing to the tunability of their special structural features and their potential ferroelectrics property. Ferroelectric materials that exhibit reversible electric polarization in response to an external electric field have found many applications such as nonvolatile memory storage, electronics and optics. The freezing of a certain functional group at low temperature forces significant orientational motions of the guest molecules and thus induces the formation of the ferroelectric phase. (Fu *et al.*, 2009; Zhang *et al.*, 2010; Zhang *et al.*, 2008;Ye *et al.*, 2006).

The asymmetric unit of the title compound is shown in Fig1, which consists of one (C_10_H_6_O_6_S_2_)^2-^ anion and one 2C_4_H_6_N_2_.4H2O.Co(II) molecule-based cation.The title complex crystallizes in monoclinic P 21/n space group, the whole compound shall be stable thanks to the numerous hydrogen bonds formed in molecules, such as the N—H···O and the O—H···O bonds. The length of N—H···O is 2.06 Å, while the length of O—H···O hydrogen bonds ranges from 1.878 to 2.205 Å. Further details about the hydrogen bonds are listed in Table 1.

### Experimental

Co(CH_3_COO^-^)_2_.4H_2_O (4.96 g, 20 mmol) mixed with K_2_CO_3_ (2.76, 20 mmol) were dissolved into 15 ml distilled water under stirring for 5 minutes, and turbid liquid was filtered,and then CoCO_3_ was obtained in about 90% yield. CoCO_3_(1.19, 10 mmol) were dissolved into solution containing 2.88 g 1,5-naphthalene disulfonic acid under stirring for 5 minutes, and then 2-methylimidazole (3.28 g, 40 mmol) were added to the solution.At last, the solution was filtered, then tansparent solution was located in a quiet and clean place, block pink crystals suitable for X-ray diffraction were obtained in about 78% yield after two days and filtered and washed with distilled water.

### Refinement

H atoms bound to carbon and nitrogen were placed at idealized positions [C—H = 0.93–0.96 Å and N—H = 0.86 Å] and allowed to ride on their parent atoms with *U*_iso_ fixed at 1.2 *U*_eq_(C,*N*). The hydrogen atoms from water molecules were added from a difference map, and the length of O—H bonds was fixed to 0.84Å with a deviation of 0.01 Å.

### Crystal data

**Table:**

[Co(C_4_H_6_N_2_)_2_(H_2_O)_4_](C_10_H_6_O_6_S_2_)	*F*(000) = 602
*M**_r_* = 581.48	*D*_x_ = 1.618 Mg m^−^^3^
Monoclinic, *P*2_1_/*n*	Mo *K*α radiation, λ = 0.71073 Å
Hall symbol: -P 2yn	Cell parameters from 3450 reflections
*a* = 8.0260 (16) Å	θ = 6.2–55.3°
*b* = 12.923 (3) Å	µ = 0.96 mm^−^^1^
*c* = 11.658 (2) Å	*T* = 293 K
β = 99.27 (3)°	Block, pink
*V* = 1193.5 (4) Å^3^	0.3 × 0.3 × 0.2 mm
*Z* = 2	

### Data collection

**Table:**

Rigaku Mercury CCD diffractometer	2729 independent reflections
Radiation source: fine-focus sealed tube	2558 reflections with *I* > 2σ(*I*)
graphite	*R*_int_ = 0.031
ω scans	θ_max_ = 27.5°, θ_min_ = 3.0°
Absorption correction: multi-scan (*CrystalClear*; Rigaku, 2005)	*h* = −10→10
*T*_min_ = 0.489, *T*_max_ = 1.000	*k* = −16→16
12041 measured reflections	*l* = −15→15

### Refinement

**Table:**

Refinement on *F*^2^	Secondary atom site location: difference Fourier map
Least-squares matrix: full	Hydrogen site location: inferred from neighbouring sites
*R*[*F*^2^ > 2σ(*F*^2^)] = 0.028	H atoms treated by a mixture of independent and constrained refinement
*wR*(*F*^2^) = 0.073	*w* = 1/[σ^2^(*F*_o_^2^) + (0.0283*P*)^2^ + 0.6244*P*] where *P* = (*F*_o_^2^ + 2*F*_c_^2^)/3
*S* = 1.09	(Δ/σ)_max_ = 0.001
2729 reflections	Δρ_max_ = 0.30 e Å^−^^3^
177 parameters	Δρ_min_ = −0.27 e Å^−^^3^
4 restraints	Extinction correction: *SHELXL97* (Sheldrick, 2008), Fc^*^=kFc[1+0.001xFc^2^λ^3^/sin(2θ)]^-1/4^
Primary atom site location: structure-invariant direct methods	Extinction coefficient: 0.0260 (17)

### Special details

**Table:**

Geometry. All e.s.d.'s (except the e.s.d. in the dihedral angle between two l.s. planes) are estimated using the full covariance matrix. The cell e.s.d.'s are taken into account individually in the estimation of e.s.d.'s in distances, angles and torsion angles; correlations between e.s.d.'s in cell parameters are only used when they are defined by crystal symmetry. An approximate (isotropic) treatment of cell e.s.d.'s is used for estimating e.s.d.'s involving l.s. planes.
Refinement. Refinement of *F*^2^ against ALL reflections. The weighted *R*-factor *wR* and goodness of fit *S* are based on *F*^2^, conventional *R*-factors *R* are based on *F*, with *F* set to zero for negative *F*^2^. The threshold expression of *F*^2^ > σ(*F*^2^) is used only for calculating *R*-factors(gt) *etc*. and is not relevant to the choice of reflections for refinement. *R*-factors based on *F*^2^ are statistically about twice as large as those based on *F*, and *R*- factors based on ALL data will be even larger.

### Fractional atomic coordinates and isotropic or equivalent isotropic displacement parameters (Å^2^)

**Table:**

	*x*	*y*	*z*	*U*_iso_*/*U*_eq_	
C1	1.0422 (2)	0.82564 (13)	0.59914 (16)	0.0329 (4)	
H1A	1.0320	0.7642	0.6389	0.039*	
C2	0.91065 (19)	0.89391 (12)	0.58123 (13)	0.0237 (3)	
C3	0.92275 (18)	0.98928 (11)	0.52102 (14)	0.0221 (3)	
C4	0.7893 (2)	1.06210 (13)	0.50084 (16)	0.0304 (4)	
H4C	0.6886	1.0486	0.5278	0.036*	
C5	0.8068 (2)	1.15174 (14)	0.44245 (18)	0.0376 (4)	
H5C	0.7178	1.1986	0.4299	0.045*	
C6	0.1443 (2)	1.11040 (14)	0.80184 (15)	0.0351 (4)	
H6B	0.0598	1.1600	0.7984	0.042*	
C7	0.2652 (3)	1.10886 (16)	0.73404 (17)	0.0411 (4)	
H7B	0.2799	1.1559	0.6761	0.049*	
C8	0.2985 (2)	0.97570 (14)	0.85354 (17)	0.0349 (4)	
C9	0.3757 (3)	0.88003 (18)	0.9091 (2)	0.0561 (6)	
H9A	0.3124	0.8573	0.9677	0.084*	
H9B	0.4901	0.8940	0.9442	0.084*	
H9C	0.3746	0.8269	0.8515	0.084*	
Co1	0.0000	1.0000	1.0000	0.02367 (11)	
H4	0.217 (4)	0.8773 (10)	1.141 (2)	0.074 (9)*	
H5	0.233 (3)	0.9781 (17)	1.1808 (15)	0.058 (8)*	
H6	0.118 (3)	1.169 (2)	1.1307 (16)	0.072 (9)*	
H7	0.006 (2)	1.2076 (11)	1.0401 (18)	0.039 (6)*	
N1	0.3618 (2)	1.02391 (14)	0.76803 (14)	0.0405 (4)	
H1B	0.4490	1.0044	0.7394	0.049*	
N2	0.16477 (19)	1.02694 (11)	0.87765 (13)	0.0310 (3)	
O1	0.05471 (19)	1.15356 (9)	1.06915 (12)	0.0378 (3)	
O2	0.20133 (17)	0.94019 (10)	1.12236 (12)	0.0356 (3)	
O3	0.60123 (15)	0.83245 (10)	0.52393 (11)	0.0341 (3)	
O4	0.66384 (16)	0.94058 (10)	0.69426 (11)	0.0378 (3)	
O5	0.76184 (17)	0.76414 (10)	0.70195 (12)	0.0396 (3)	
S1	0.72008 (5)	0.85486 (3)	0.62836 (3)	0.02460 (12)	

### Atomic displacement parameters (Å^2^)

**Table:**

	*U*^11^	*U*^22^	*U*^33^	*U*^12^	*U*^13^	*U*^23^
C1	0.0290 (8)	0.0262 (8)	0.0442 (10)	0.0024 (7)	0.0079 (7)	0.0075 (7)
C2	0.0199 (7)	0.0238 (7)	0.0278 (8)	−0.0024 (6)	0.0052 (6)	−0.0018 (6)
C3	0.0178 (7)	0.0225 (7)	0.0259 (7)	−0.0013 (5)	0.0031 (6)	−0.0029 (6)
C4	0.0197 (7)	0.0304 (8)	0.0426 (9)	0.0035 (6)	0.0097 (7)	0.0026 (7)
C5	0.0253 (8)	0.0317 (9)	0.0572 (12)	0.0098 (7)	0.0111 (8)	0.0098 (8)
C6	0.0412 (10)	0.0322 (9)	0.0334 (9)	−0.0028 (7)	0.0111 (8)	0.0004 (7)
C7	0.0494 (11)	0.0433 (11)	0.0333 (9)	−0.0123 (9)	0.0142 (8)	−0.0024 (8)
C8	0.0334 (9)	0.0347 (9)	0.0394 (10)	−0.0020 (7)	0.0148 (8)	−0.0073 (7)
C9	0.0531 (13)	0.0455 (12)	0.0764 (16)	0.0166 (10)	0.0304 (12)	0.0034 (11)
Co1	0.02407 (17)	0.02192 (17)	0.02595 (17)	0.00133 (11)	0.00684 (12)	0.00013 (11)
N1	0.0364 (9)	0.0496 (9)	0.0405 (9)	−0.0068 (7)	0.0215 (7)	−0.0112 (7)
N2	0.0325 (8)	0.0285 (7)	0.0346 (8)	−0.0008 (6)	0.0134 (6)	−0.0015 (6)
O1	0.0499 (8)	0.0237 (6)	0.0364 (7)	0.0016 (5)	−0.0035 (6)	−0.0006 (5)
O2	0.0363 (7)	0.0313 (7)	0.0365 (7)	0.0043 (5)	−0.0022 (5)	−0.0040 (5)
O3	0.0273 (6)	0.0388 (7)	0.0354 (7)	−0.0091 (5)	0.0028 (5)	−0.0059 (5)
O4	0.0373 (7)	0.0389 (7)	0.0416 (7)	−0.0062 (5)	0.0197 (6)	−0.0141 (6)
O5	0.0402 (7)	0.0370 (7)	0.0431 (7)	−0.0047 (6)	0.0115 (6)	0.0119 (6)
S1	0.0228 (2)	0.0253 (2)	0.0269 (2)	−0.00531 (14)	0.00757 (15)	−0.00294 (14)

### Geometric parameters (Å, °)

**Table:**

C1—C2	1.366 (2)	C8—C9	1.484 (3)
C1—C5^i^	1.406 (2)	C9—H9A	0.9600
C1—H1A	0.9300	C9—H9B	0.9600
C2—C3	1.429 (2)	C9—H9C	0.9600
C2—S1	1.7795 (16)	Co1—O2^ii^	2.1215 (14)
C3—C4	1.417 (2)	Co1—O2	2.1215 (14)
C3—C3^i^	1.432 (3)	Co1—N2	2.1242 (15)
C4—C5	1.362 (2)	Co1—N2^ii^	2.1242 (15)
C4—H4C	0.9300	Co1—O1^ii^	2.1603 (13)
C5—C1^i^	1.406 (2)	Co1—O1	2.1603 (13)
C5—H5C	0.9300	N1—H1B	0.8600
C6—C7	1.347 (3)	O1—H6	0.837 (10)
C6—N2	1.387 (2)	O1—H7	0.843 (9)
C6—H6B	0.9300	O2—H4	0.845 (10)
C7—N1	1.365 (3)	O2—H5	0.844 (10)
C7—H7B	0.9300	O3—S1	1.4498 (13)
C8—N2	1.329 (2)	O4—S1	1.4605 (12)
C8—N1	1.343 (2)	O5—S1	1.4596 (13)
			
C2—C1—C5^i^	120.04 (16)	O2—Co1—N2	91.27 (6)
C2—C1—H1A	120.0	O2^ii^—Co1—N2^ii^	91.27 (6)
C5^i^—C1—H1A	120.0	O2—Co1—N2^ii^	88.73 (6)
C1—C2—C3	121.33 (14)	N2—Co1—N2^ii^	180.000 (1)
C1—C2—S1	116.83 (12)	O2^ii^—Co1—O1^ii^	89.81 (5)
C3—C2—S1	121.73 (11)	O2—Co1—O1^ii^	90.19 (5)
C4—C3—C2	123.03 (14)	N2—Co1—O1^ii^	90.63 (6)
C4—C3—C3^i^	119.19 (17)	N2^ii^—Co1—O1^ii^	89.37 (6)
C2—C3—C3^i^	117.78 (17)	O2^ii^—Co1—O1	90.19 (5)
C5—C4—C3	120.77 (15)	O2—Co1—O1	89.81 (5)
C5—C4—H4C	119.6	N2—Co1—O1	89.37 (6)
C3—C4—H4C	119.6	N2^ii^—Co1—O1	90.63 (6)
C4—C5—C1^i^	120.88 (16)	O1^ii^—Co1—O1	180.0
C4—C5—H5C	119.6	C8—N1—C7	108.86 (16)
C1^i^—C5—H5C	119.6	C8—N1—H1B	125.6
C7—C6—N2	109.89 (17)	C7—N1—H1B	125.6
C7—C6—H6B	125.1	C8—N2—C6	105.61 (15)
N2—C6—H6B	125.1	C8—N2—Co1	132.19 (13)
C6—C7—N1	105.70 (17)	C6—N2—Co1	122.19 (12)
C6—C7—H7B	127.2	Co1—O1—H6	127.2 (19)
N1—C7—H7B	127.2	Co1—O1—H7	124.0 (15)
N2—C8—N1	109.93 (17)	H6—O1—H7	109 (2)
N2—C8—C9	128.11 (18)	Co1—O2—H4	126 (2)
N1—C8—C9	121.95 (18)	Co1—O2—H5	115.3 (18)
C8—C9—H9A	109.5	H4—O2—H5	110 (3)
C8—C9—H9B	109.5	O3—S1—O5	113.01 (8)
H9A—C9—H9B	109.5	O3—S1—O4	112.08 (8)
C8—C9—H9C	109.5	O5—S1—O4	111.17 (8)
H9A—C9—H9C	109.5	O3—S1—C2	106.22 (8)
H9B—C9—H9C	109.5	O5—S1—C2	106.38 (8)
O2^ii^—Co1—O2	180.0	O4—S1—C2	107.53 (7)
O2^ii^—Co1—N2	88.73 (6)		

Symmetry codes: (i) −*x*+2, −*y*+2, −*z*+1; (ii) −*x*, −*y*+2, −*z*+2.

### Hydrogen-bond geometry (Å, °)

**Table:**

*D*—H···*A*	*D*—H	H···*A*	*D*···*A*	*D*—H···*A*
O2—H4···O5^iii^	0.85 (1)	1.97 (1)	2.815 (2)	174 (3)
O2—H5···O4^iv^	0.84 (1)	1.88 (1)	2.7149 (19)	171 (3)
O1—H6···O5^iv^	0.84 (1)	2.21 (1)	3.026 (2)	167 (3)
O1—H7···O3^v^	0.84 (1)	1.92 (1)	2.7661 (18)	179 (2)
N1—H1B···O4	0.86	2.06	2.906 (2)	170

Symmetry codes: (iii) *x*−1/2, −*y*+3/2, *z*+1/2; (iv) −*x*+1, −*y*+2, −*z*+2; (v) −*x*+1/2, *y*+1/2, −*z*+3/2.
